# Horse genetics, archaeology, and the beginning of riding

**DOI:** 10.1126/sciadv.ady7336

**Published:** 2026-05-13

**Authors:** David Anthony, Martin Trautmann, Volker Heyd

**Affiliations:** ^1^ Hartwick College, Oneonta, NY 13820, USA.; ^2^ Harvard University, Cambridge, MA 02138, USA.; ^3^ Department of Cultures/Archaeology, University of Helsinki, Helsinki, Finland.

## Abstract

Recent papers argued that the domestication of horses can be equated with the appearance of favorable genetic mutations that are first evident in individuals in the DOM2 clade dated about ∼2200–2100 BCE. We challenge the idea that this genetic shift alone defines domestication. Evidence from archaeology, ancient DNA, osteology, and other disciplines shows that horses from multiple genetic backgrounds (DOM1, DOM2, and, as we suggest here, DOM3) were managed, milked, and ridden long before 2200 BCE. Yamnaya groups (∼3200–2600 BCE) rode DOM2 horses—the direct ancestors of modern domestic stock—while incorporating them into diets, rituals, and mobility systems. Selection for traits linked to endurance and temperament began centuries earlier. Rather than a sudden breakthrough, domestication was a protracted, regionally varied process whose transformative effects on human mobility and social organization began as early as the fourth, if not the fifth millennium BCE, and set the stage for later DOM2 dominance.

## Teaser

aDNA, archaeozoology, and archaeology reveal that humans tamed and rode horses long before full domestication transformed history.

## INTRODUCTION

**Fig. 1.**
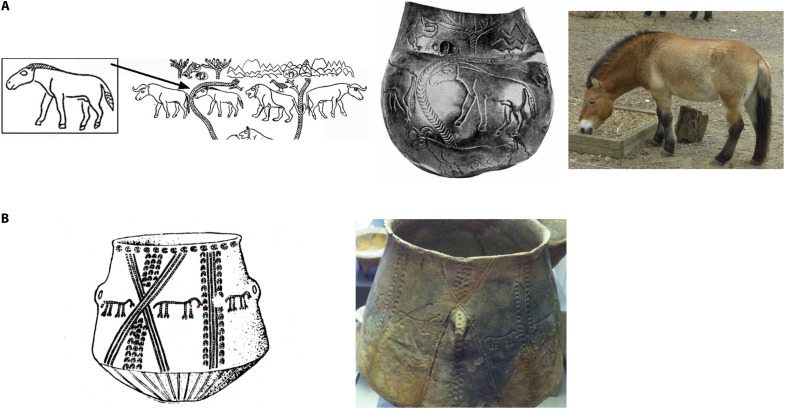
Early horse representations in the steppes. (**A**) The earliest clear image of a steppe horse, embossed on a silver cup from the Maikop-Oshad chieftain’s grave, Russia (*107*) dated 3520–3350 calBCE at 2σ (4645 ± 23 BP; OxA-X-3106-13), located at the ecotone between the North Caucasus Kuban piedmont and the Volga-Don steppes where modern DOM2 horses evolved, so probably a horse of the DOM2 clade. The large head, stiff mane, thick neck, and partly tufted tail is like a modern *E. ferus przewalskii* (*E. przewalksii*) of the DOM1 clade, picture on the right. Drawings from (*70*), figure 12.10, used with permission from Princeton University Press, from “The Horse, the Wheel, and Language: How Bronze-Age Riders from the Eurasian Steppes Shaped the Modern World,” by D. W. Anthony, book, first edition, 2010 (permission conveyed through Copyright Clearance Center, Inc.); (*108*), figure 3, reproduced with permission from the publisher; photo by D.A. (**B**) A ring of six horses around a vessel from Usatovo in the Dnister steppes, Ukraine, dated ∼3300–2800 BCE; photo by D.A., taken in the State Historical Museum, Moscow; drawing from (*109*), figure 33. BP, before present.

The domestication of horses and the beginning of horseback riding profoundly changed human history, yet our understanding of both the domestication process and of early riding is contested (Fig. 1). Five papers, two in *Nature* and one in *Science* by the equine genetics team led by Orlando (*1*–*3*), and two co-authored by Taylor in *Science Advances* (*4*) and *Nature Scientific Reports* (*5*), recently challenged widely accepted views on the antiquity of horse domestication and riding. The Taylor papers argued that the effective domestication of horses happened after ∼2200–2100 BCE, contemporary with the invention of chariots, and widespread riding began even later, after ∼1500 BCE. The Orlando team recognized earlier efforts to tame horses but concluded that any riding that might have occurred earlier had only limited effects on human mobility before genetic mutations culminated in calmer, higher-endurance horses of the DOM2 lineage dated after ∼2200–2100 BCE. While giving some nuance to the process and attempting to distinguish between “earlier taming” and “later domesticating,” they still regard the use of the term “domesticated” solely for the widespread appearance of horses with changes in the *GSDMC* locus, perhaps easing back pain in horses, increasing their endurance when ridden, and possibly improving overall body conformation; and in the *ZFPM1* locus related to fear and anxiety, perhaps making horses calmer around humans (again an adaptation to prior handling). After ∼2200–2100 BCE, DOM2 horses with these favorable mutations quickly spread across Europe, Anatolia, the Near East, and Central Asia, replacing earlier varieties, and this important event was said to represent the effective beginning of domestication, riding, and horse-based mobility (*1*–*3*). They documented a “domestication bottleneck” that began ∼2700 BCE, when intensified selection began on these loci, which they equated with “early taming,” but reserved the term domestication for the end of the bottleneck: “…breeding within close genealogical kin started with the earliest stages of DOM2 domestication, ~2,200 BCE” [Librado *et al.* (*2*), Supplementary information, p. 27].

Horses associated with the Yamnaya culture (∼3200–2600 BCE) were specifically excluded [Librado *et al.* (*2*), p. 821] from “…any significant involvement…in the Yamnaya-related or earlier human migrations from the steppe.” The expansion of steppe ancestry in human populations across Central and Western Eurasia associated with the movement of people archaeologically identified as Yamnaya (*6*–*8*) was facilitated, according to Hosek *et al.* (*4*), only by the wagons that were buried in Yamnaya graves, not by riding. Hosek *et al.* (*4*) asserted, contrary to published evidence, that there is “no evidence of the domestic horse” (p. 2) in any Yamnaya or Afanasievo (Yamnaya’s eastern offshoot in the Altai Mountains) archaeological context, and that (p. 6) “...horse genomics now demonstrates that there is little or no connection between horses found at Yamnaya sites and the ancestors of modern domestic horses, and genomic indicators of domestication do not emerge in the Black Sea region until well into the third millennium BCE.” Last, Librado *et al.* (*2*) introduced a measure of horse generation times that demonstrated “…that new practices of DOM2 reproductive control, aimed at faster productivity, emerged by the late third millennium BCE, and were a prerequisite to early DOM2 breeding and adoption of widespread horse-based mobility.” The same phenomenon of reduced generation times was, however, also observed for the DOM1 later, fourth millennium BCE horses of Botai, and the slightly later (~3000 BCE) horses of the site of Borly4 near the Irtysh river in Kazakhstan, supporting the existence of earlier human horse management at these sites (*9*).

**Fig. 2.**
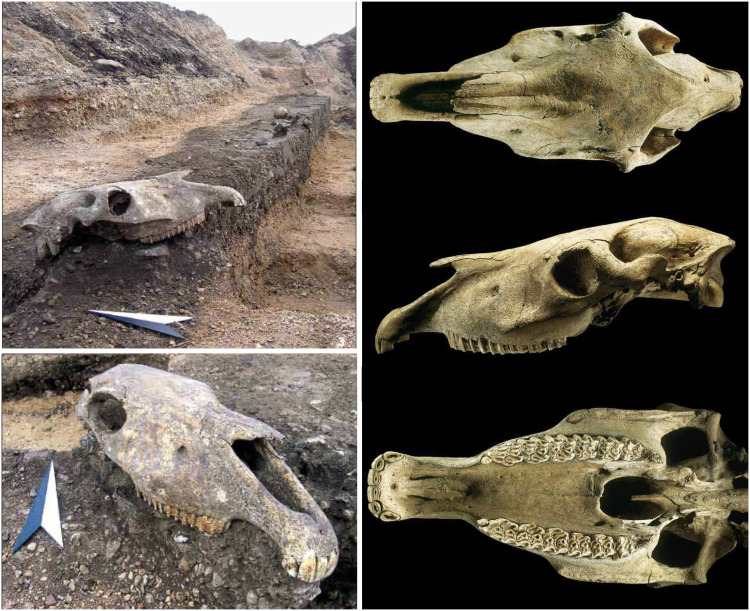
The skull of the Salzmünde tobiano horse, Germany. Skull, dated 3368–3101 calBCE at 2σ, as found in situ and from various directions. Photos from (*110*), figures 3 to 7: original photos by T. Schunke (figure 3), H. Arnold (figure 4), and J. Lipták (figures 5 to 7), all from the State Office for Heritage Management and Archaeology Saxony-Anhalt; reproduced with permission from the publisher.

We argue, in agreement with Outram (*9*) and Kanne (*10*) that the aggressive expansion of DOM2 horses after ∼2200–2100 BCE was not preordained in the genetically more diverse domesticated horse herds of ∼3500–3000 BCE, when horses in three distinct genetic populations, DOM1, DOM2, and, as we suggest here, DOM3, were managed and ridden without much regard for their genetic ancestry. Archaeological and genetic evidence indicates that horse domestication was a long process, not an event (*9*, *11*). Horses were used and managed across Eurasia in the fourth millennium BCE and already were shifting away from the “prey” pole on the pathway to domestication (*11*). Before the domestication bottleneck in the DOM2 clade, horses of different genetic ancestries might have appeared similar, according to pictorial evidence as in Fig. 1, and were managed similarly according to direct evidence for milking and riding in both the DOM1 and DOM2 populations. The gene for tobiano spotting, a variety of pinto coloring found only in domesticated horses (*12*), appeared before 3100 BCE in both the DOM1 (Botai) and our suggested DOM3 clade (at Salzmünde, Germany; Fig. 2), dated 3368–3101 calibrated BCE (calBCE) at 2σ (=95.4% probability; 4552 ± 29 before present (BP); KIA-31406). This perspective revives the interesting question of why different horse populations and other equids, including asses and onagers in the Near East, began to be exploited as transport animals at about the same time between ∼3500 and 3000 BCE (*13*) and why the modern DOM2 mutations appeared in the DOM2 clade only.

In this response, we defend published lines of evidence suggesting that horse domestication and riding began before 2200–2100 BCE. Librado *et al.*’s (*1*) argument that Yamnaya people did not ride horses depended largely on their interpretation of the genetic ancestry of four Corded Ware culture (CWC) horses from a multicultural ritual site in Germany (Hohler Stein at Schwabthal, in detail below), from which they derived conclusions about Yamnaya horse riding in the steppes. The genetic ancestry of these CWC horses was reanalyzed by Maier *et al.* (*14*) using analytical tools that gave different results; they warned that no firm conclusion on CWC horse ancestry was possible with the available data and methods. A reanalysis of the same CWC horse dataset by Librado *et al.* (*2*) using Maier *et al.*’s tools was argued to support their original interpretation: that conclusion remains unchallenged. Both Librado *et al.* (*2*) and Hosek *et al.* (*4*) attempted to set aside Wilkin *et al.*’s (*15*) proteomic evidence for equine milk consumption by Yamnaya people, and Hosek *et al.* (*4*) challenged Trautmann *et al.*’s (*16*) identification of skeletal changes associated with habitual horse riding in Yamnaya individuals, both involving authors of this paper. Last, Librado *et al.* (*2*) did not discuss a large literature on ∼3500–3000 BCE horse domestication in Central Europe, although they published samples relevant to that debate. By ignoring the evidence for horse domestication dated between ∼3500 and 3000 BCE in Europe (DOM3 as defined here) and dismissing the Yamnaya and earlier evidence in the Pontic-Caspian steppes (DOM2), they could characterize the ∼3500–3100 BCE evidence for horse management at Botai (DOM1) as isolated and unimportant, while we argue that it was part of a widespread shift in human control over equids.

Evidence cited below suggests that the Yamnaya people ate horses in some places, milked them in others, and occasionally placed their body parts in human graves and in the fillings of at least five kurgans in Southeast Europe, and that some Yamnaya people habitually rode horses. Their DOM2-clade horses were the direct genetic ancestors of all modern domesticated horses, contributing 95% of the ancestry of DOM2 horses dated after ∼2200–2100 BCE according to Librado *et al.* (*1*). The migration of Yamnaya and Yamnaya-related steppe people into Europe that began ∼3100 BCE had the largest effect on European human genomes of any demographic event in the last 5000 years and was part of a movement out of the steppes that spanned 5000 km across Eurasia between ∼3200 and 2600 BCE—an unprecedented burst of long-distance mobility and a possible vector for the spread of Indo-European languages, plausibly aided by horse riding (*6*–*8*, *16*, *17*).

## RESULTS

### A review of evidence for horse domestication in three horse populations

**Fig. 3.**
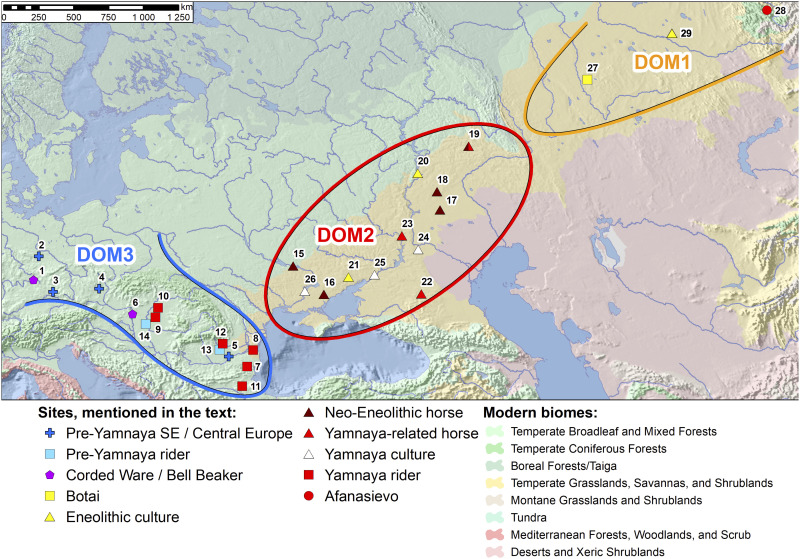
The general distributions of DOM1, DOM2, and DOM3 horses at ∼3500–3000 BCE, and locations of the main sites mentioned in the text. Pre-Yamnaya DOM3 horse aDNA came from sites marked with a cross; Yamnaya and pre-Yamnaya humans with rider syndrome, as listed in Trautmann *et al.* (*16*), were from graves marked with a square; DOM2 Steppe Neolithic and Eneolithic horse aDNA samples (NEONCAS group) were from #15 to #19; DOM2 Steppe Yamnaya and related horses (CPONT and TURG groups) were from #19 and #22 to #23; the genetic ancestry of number #28 is unknown. Geographic Information System (GIS)-based map created by L. Vyazov. Sites: 1, Schwabthal-Hohler Stein; 2, Salzmünde; 3, Cham; 4, Stránská skála; 5, Pietrele; 6, Csepel; 7, Vetrino; 8, Medgidia; 9, Dévaványa; 10, Balmazújváros; 11, Malomirovo; 12, Strejnicu; 13, Blejoi; 14, Csongrád; 15, Deriivka; 16, Semenivka; 17, Varfolomeevka; 18, Oroshaemoe; 19, Turganik; 20, Khvalynsk; 21, Razdol’noe; 22, Aygurskii; 23, Repin; 24, Tsatsa; 25, Kriviansky IX; 26, Mykhailivka; 27, Botai; 28, Nizhnyaya Sooru; 29, Borly4. SE, Southeast.

Librado *et al.* (*1*, *2*) and Gaunitz *et al.* (*18*) analyzed an invaluable array of horse genomes that will be an essential resource for all future research on horse domestication and genetics. They recognized at least three major regional populations by their distinct genetics at ∼3500–3000 BCE (Fig. 3): DOM1 in the Central Asian steppes east of the Ural Mountains, including the domesticated horses of Botai; DOM2 in the European or Pontic-Caspian steppes west of the Ural Mountains and north of the Black and Caspian Seas, including the horses of the Yamnaya culture and earlier Neolithic-Eneolithic steppe horses; and an unnamed European-Anatolian lineage called DOM3 here. Librado *et al.* (*2*), who briefly described this grouping, did not use an acronym for it. They did not discuss the possibility of domestication within this population, so a DOM label perhaps seemed unjustified.

### DOM1: Archaeological and osteological evidence for domestication in Botai horses

**Fig. 4.**
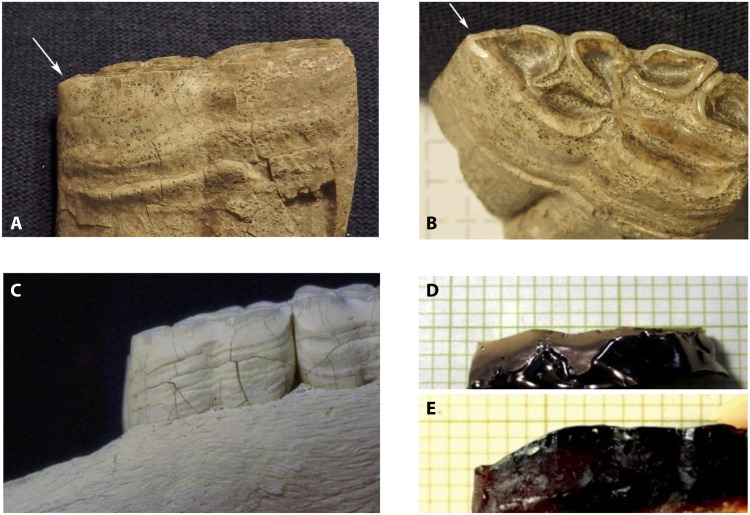
Bit wear on horse P_2_’s at Botai, Kazakhstan. (**A**) Arrow points to a 4-mm facet on the mesial (front) corner of a P_2_ from Botai specimen 43. (**B**) The facet from the front. (**C**) Never-bitted Nevada mustang P_2_ with no wear facet. (**D**) Side view of an epoxy replica from a high-resolution cast of the P_2_ of a never-bitted modern horse ridden experimentally with only a horsehair rope bit for 150 hours exhibiting a wear facet measuring 2.5 mm (⅒ inch). (**E**) Epoxy replica of a Botai P_2_ with a 6-mm facet, shaped like the experimentally created facet in (D). ⅒ inch ruled plastic sheet against (D) and (E); all photos by D.A.

Most observers (*10*, *11*, *19*) accepted that horses were domesticated between ∼3500 and 3100 BCE at Botai, Kazakhstan and related horse-keeping settlements in the steppes east of the Ural Mountains (*9*, *20*). The Botai people were Central Asian hunter-gatherers who domesticated horses locally and became specialized horse keepers, hunters, and pastoralists, with no other domesticated animals except dogs. At Botai, horses arguably were corralled, milked, and poleaxed; their manure was gathered and discarded in pits; they were butchered in the settlement; and they constituted 99% of the fauna consumed (*9*, *20*, *21*). That some Botai horses (Fig. 4) were ridden might be indicated by wear from rope bits on their lower second premolars (P_2_’s) (*22*, *23*). Anthony and Brown (*22*, *24*) compared 73 P_2_’s from modern horses bitted with metal bits (at the Cornell University Veterinary School) to eight P_2_’s from four horses bitted experimentally with organic bits and ridden for 150 hours each (at the State University of New York at Cobleskill), and to 177 P_2_’s from never-bitted Pleistocene horses (at the University of Florida) and Nevada mustangs (from the Bureau of Land Management), showing that a facet on the front (paraconid) cusp of the P_2_ measuring ≥3 mm was significantly associated with bitted horses (*22*, *24*). Of 19 measurable P_2_’s at Botai, five had facets of 3 to 6 mm, which, by their similarity to our experimentally produced rope bit wear, were consistent with being made by a rope bit containing embedded dirt and grit derived from pasture that was the actual agent of wear. Outram and Bendrey *et al.* (*20*) identified other Botai dental pathologies arguably caused by bits. Taylor and Barrón-Ortiz (*5*) dismissed all this evidence, regarding the Botai horses as victims of mass intercept hunting, reviving a hunted-wild-population interpretation advanced earlier (*21*). Outram and Orlando rebutted Taylor’s critique in Outram *et al.* (*23*). DOM1 horses survive today as *E. ferusprzewalskii* (*E. przewalskii*), feral descendants of the domesticated horses of Botai.

### DOM2: Archaeological and osteological evidence for domestication in Yamnaya horses

Domesticated horses usually are also assigned to the partly contemporary Yamnaya culture dated ∼3200–2600 BCE in the Pontic-Caspian steppes, west of the Ural Mountains (*25*–*28*). Skeletal pathologies associated with horse riding occurred in Yamnaya and pre-Yamnaya humans in Southeast Europe (*16*), and peptides from horse milk were identified in the dental calculus of two Yamnaya individuals (*15*). Horse bones appeared occasionally in Yamnaya-related graves, in the fillings of their kurgans, and consistently in Yamnaya settlements, but usually in numbers too small for zoological studies of size or type (*25*). At Mykhailivka on the lower Dnipro, the largest excavated Yamnaya settlement, with 51,151 identified animal bones probably representing feasting events (*28*), cattle were 60% of bones and 45% of the minimum number of individuals (MNI), followed by sheep-goats (30% bones/34% MNI) and horses (10% bones/18% MNI) (*25*, *29*, *30*). Horse fats were detected on 37% of the ceramic sherds from Mykhailivka level II (early Yamnaya) analyzed for lipid residues [(*31*), p. 6], so it might have been more important in the diet than the bone count suggests. The earliest-dated person [3635–3383 calBCE at 2σ (4755 ± 25 BP; PSUAMS-10750)] exhibiting the typical Yamnaya genome was a female (I32534) who left a loose tooth at Mykhailivka level II (*8*). About 10% of the rim sherds at Mykhailivka level II were from Repin-style pots (*32*), defined by the ceramics at Repin on the Don River, an early or proto-Yamnaya settlement near a natural bluff still called the Mare’s Head, where horses were 80% of the fauna in both the initial report and in a small re-excavation in the 1980s, with the bones of sheep/goat, cattle, and red deer in smaller percentages (*28*, *32*). In the Yamnaya kurgan cemetery at Tsatsa in the North Caspian steppes, 40 horse heads were buried with a male in the late Yamnaya/early Katacombnaya kurgan 1, grave 12, dated by pottery and artifacts to ∼2800–2600 BCE (*8*, *25*). Anthony (*28*) published tables with numbers of horse bones found in graves in different Yamnaya regions, with the Volga-Ural region having the most frequent horse bones, appearing there in about 25% of Yamnaya graves containing animal bones.

Contrary to Hosek *et al.*’s (*4*) claim, there is substantial evidence for the incorporation of horse meat and milk in the Yamnaya diet, and horses were offered with sheep and cattle in Yamnaya funeral rituals. Already in the Eneolithic steppes, horses were buried with humans, cattle, and sheep, and horse-head maces were important political symbols, as discussed below. In Yamnaya contexts, pathologies associated with riding appeared in multiple humans (see below). The mobile form of multispecies pastoralism introduced in the Pontic-Caspian steppes with the early Yamnaya culture ∼3200 BCE arguably required domesticated horses, particularly in drier southern steppe/semi-desert regions in winter, to control large herds and to open paths in snow for sheep and cattle to graze (*27*, *28*, *33*). Librado *et al.* (*1*) found that Yamnaya horses, labeled CPONT (Caspian-Pontic) or TURG (for the Turganik site in the Volga-Ural steppes), belonged to the DOM2 clade and contributed 95% of the ancestry of DOM2 horses dated after ∼2200–2100 BCE. They showed that Yamnaya horses were the direct ancestors of all modern domesticated horses.

### DOM3: Archaeological and osteological evidence for domestication in Central and Southeast European horses

Librado *et al.* (*2*) briefly described a group rooted in the Pleistocene that apparently linked horse populations from France to Anatolia—a single land mass before the Bosporus breakthrough occurred ∼9000 BCE (*34*), isolating Anatolian horses. Indigenous European horses adapted to meadow or marsh areas within forested regions are here labeled DOM3, accepting the shared ancestry proposed by Librado *et al.* (*2*) while recognizing that additional sampling is needed. DOM3 ancestry survived into the 19th century in feral steppe horses (“tarpans”) but is neglectable in modern horses, which are descended almost entirely from DOM2 ancestors. The amount and timing of admixture between DOM2 and our DOM3 horses was a central topic for Librado *et al.* (*2*), but they did not refer to the debate on this topic among zoologists and archaeologists.

**Fig. 5.**
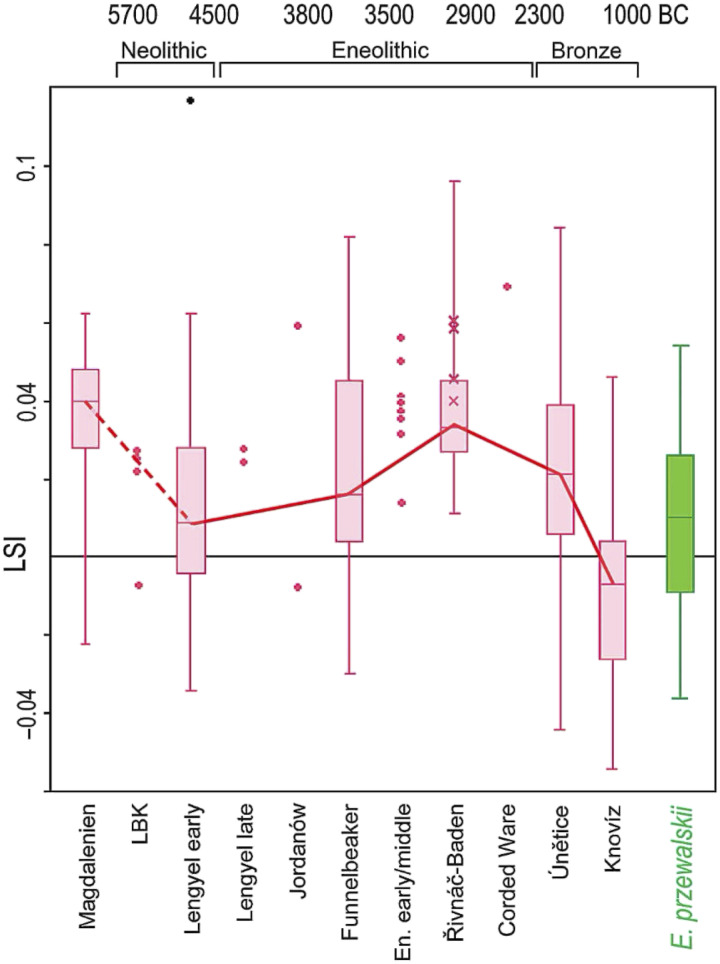
The relative size of horses (LSI) in different archaeological cultures of Czechia (in red) in comparison to today’s *E. przewalskii* (in green). The graph shows relatively significant changes in size and a widening of variability during the Eneolithic and the Bronze Age. LSI, logarithmic size index. Adapted reproduction from (*37*), figure 3, under a CC BY 4.0 international license.

Since the work of Bőkőnyi (*35*, *36*), the significance of the appearance of larger horses after ∼3500 BCE in Central and Southeast Europe has been debated in literatures, as recently reviewed by Kyselý and Peške (*37*) for Czechia, and Bozi and Szabó (*38*) and Bondár (*39*) for Hungary. Based on documented increases in horse size and size variability, often a signal of human management, the archaeozoologists Uerpmann (*40*), Glass (*41*), and Benecke (*42*) argued that the Indigenous European horses of the Salzmünde, Bernburg, and Cham cultures in Germany (our suggested DOM3) probably were domesticated well before 3100 BCE, perhaps with some admixture from larger steppe horses (now DOM2). Benecke [(*42*), p. 204] concluded that some of the larger Bernburg horses were used as pack animals or for riding. Kyselý and Peške (*37*) observed similar increases in size and variability (Fig. 5) in Baalberge-Trichterbecherkultur (TRB) and Baden horses in Czechia dated well before 3100 BCE. A horse from Salzmünde dated 3368–3101 calBCE at 2σ (4552 ± 29 BP; KIA-31406) had the gene for tobiano spotting (*12*) found only in domesticated horses and identified earliest at Botai and at Salzmünde, but probably distributed between them before ∼3100 BCE. The horses of Salzmünde were identified zoologically as domesticated (*43*).

In the Carpathian Basin and the Balkans, horse bones are known from about two dozen sites, mostly of Boleráz, Baden, and Coțofeni cultures and their equivalents further east. While horses do not form part of Baden grave assemblages, there are at least three examples of complete horse bodies buried in settlement/refuse pits (*44*). As in Central Europe, ∼3500–3000 BCE horse bone assemblages are limited in quantity, in strong contrast to some ∼2400–2000 BCE Hungarian Bell Beaker and Early Bronze Age sites. Exceptional is the site of Kiten-Urdoviza beach, a submerged settlement at the Bulgarian Black Sea coast. This site culturally belongs to the so-called Ezerovo culture and is dated to ∼3100 BCE, making it roughly contemporary with early Yamnaya. Here, apparently about 450 horse bones/fragments were recovered in an overall collection of several thousand bones, and local domestication is claimed (*45*).

These varied signals of fourth millennium BCE horse management in Central and Southeast Europe probably can be attributed to horses with mostly local European DOM3 ancestry. Genetic evidence reviewed below suggests that Pontic-Caspian steppe DOM2 horse ancestry spread into DOM3 European horse populations notably in the lower Danube valley in the fifth millennium BCE but expanded to the Carpathian Basin and beyond only gradually and in relatively small amounts before the Bell Beaker period.

### DOM2 horse management 4500–2700 BCE: Early domestication

Librado *et al.* (*1*) established that the DOM2 clade evolved in the Pontic-Caspian steppes. The early stages in the domestication process within the DOM2 clade prior to the genetic changes of ∼2200–2100 BCE have been modeled in genetics papers (*2*, *3*), but with little attention to the archaeological and cultural context of the sampled horses.

The earliest horses sampled in the DOM2 clade were from “ceramic Neolithic” hunter-gatherer sites without domesticated animals dated ∼5500 BCE in the Volga-Ural-Caspian steppes, labeled NEONCAS (Neolithic North Caspian) by Librado *et al.* (*1*). From their sixth millennium BCE archaeological contexts at Varfolomeevka and Algay in the North Caspian steppes and Turganik in the Southwest Ural steppes (*46*), most of the NEONCAS horses can be confirmed as wild because the other faunas found with them were wild (*47*).

**Fig. 6.**
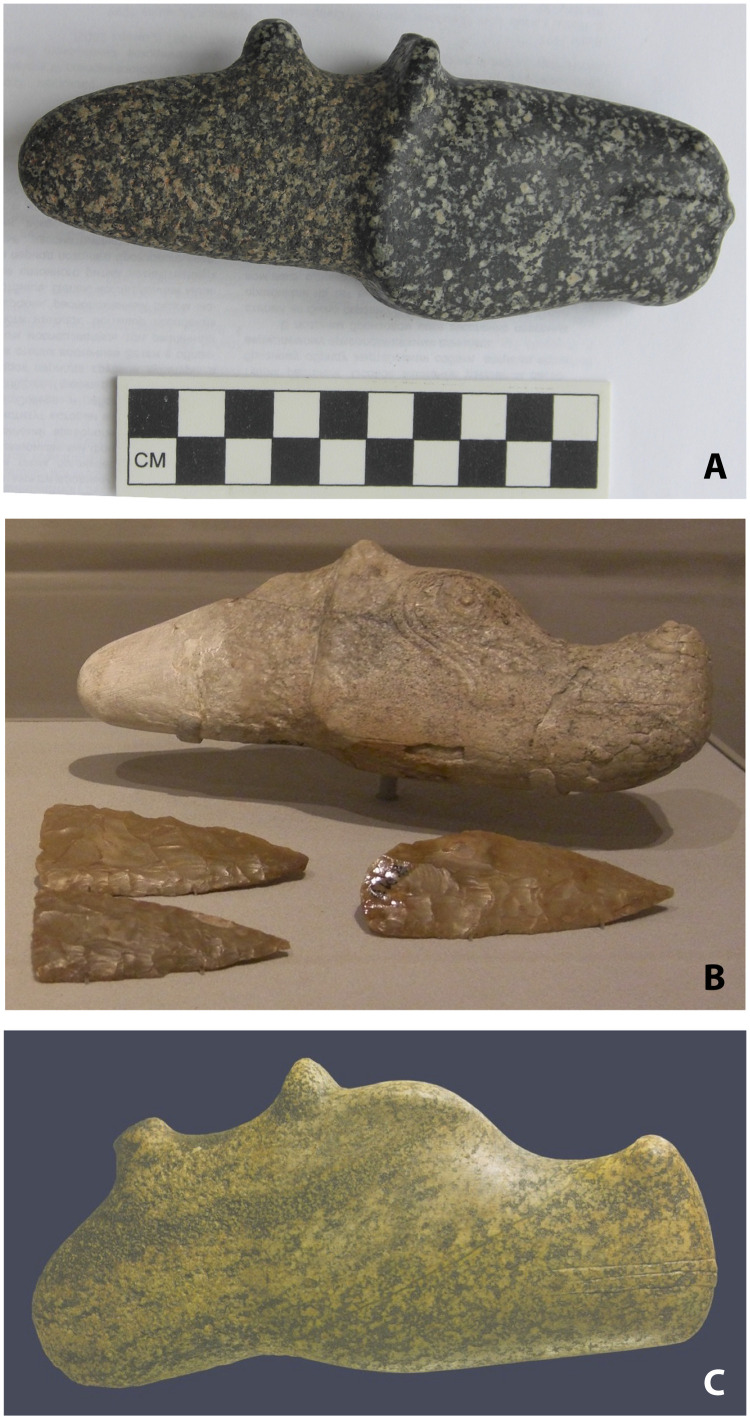
Examples of stone horse-head maces, dated to ∼4500–4200 BCE. (**A**) Suvurovo, Ukraine; photo by D.A. taken in the Odesa Museum, Ukraine. (**B**) Casimcea Romania; photo by D.A. in the museum of the Institute for the Study of the Ancient World in New York, USA. (**C**) Drama, Bulgaria, photo by the Regional Historical Museum Yambol, Bulgaria (reproduced with permission from the Museum).

Considering their broader archaeological context, domesticated cattle appeared by ∼5500 BCE [5622–5479 calBCE at 2σ (6609 ± 49 BP; Ua-42031] at Razdol’noe in the Dnipro-Azov steppes (*48*), and by ∼4700 BCE, first sheep-goat and then cattle appeared in the lower Volga steppes (*46*, *47*). Eneolithic settlement sites dated to the fifth and fourth millennia BCE such as Deriivka on the Dnipro and Varfolomievka on the lower Volga had between 20 and 55% horse bones mixed with the bones of domesticated cattle and sheep/goat [faunal tables in (*30*); reviewed in (*22*); newly obtained data in (*46*, *47*)]. In Eneolithic cemeteries at Khvalynsk and S’yezzh’ye in the middle Volga steppes dated to ∼4500–4200 BCE (*49*), horse head and hoof parts [not analyzed for ancient DNA (aDNA)] were deposited in graves with humans [analyzed for aDNA in (*8*)] and the head and hoof parts of domesticated sheep, goats, and cattle (no wild animals). These graves and others dated after ∼4500 BCE also contained bone carvings of horse images and polished stone zoomorphic mace heads (Fig. 6) shaped like horse heads (*30*). Beginning ∼4500 BCE, people in the Volga steppes began to include horses with cattle and sheep in human funeral rituals and as symbols in political-military culture, shifting them off the wild pole and toward the domesticated pole on the domestication continuum.

**Fig. 7.**
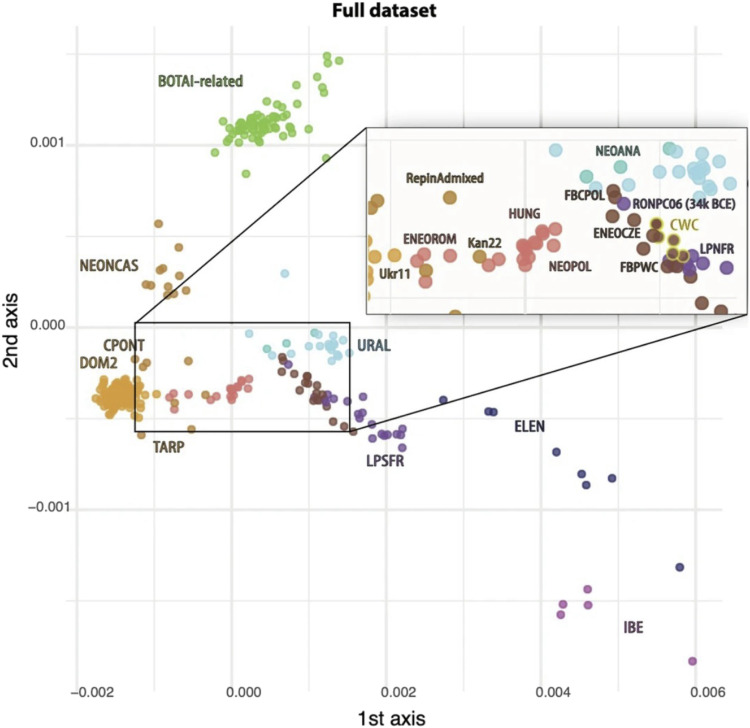
The two first dimensions of an MDS analysis. Summarizing the genomic affinities between horses in their full dataset (NEOANA, Neolithic Anatolia; FBPWC, Funnel Beaker/Pitted Ware; NEOPOL, Neolithic Poland; ENEOCZE, Eneolithic Czechia; LPNFR, Late Paleolithic North France; Ukr11, Seredni Stih at Semenivka; ENEOROM, Eneolithic Gumelnitsa Romania; HUNG, Baden and Bell Beaker horses; and URAL, Paleolithic and Mesolithic forest zone Russia). Adapted reproduction from (*2*), extended data: figure 5a, under a CC BY 4.0 international license.

Two sampled Eneolithic horses were from contexts broadly contemporary with the horse-head maces. They were different, one (Ukr11) plotting very close to DOM2 (Fig. 7) in metric multidimensional scaling (MDS) analysis and the other plotting with wild NEONCAS horses (*1*). Ukr11 came from the Seredni Stih culture occupation at Semenivka [(*50*), p. 125] in the Dnipro-Azov steppes [4315–4054 calBCE at 2σ (5340 ± 20 BP; UCIAMS-224904)]. This horse was found in stratum 3 with domesticated sheep, goats, and cattle. The other Eneolithic sample, from Oroshaemoe on the lower Volga [4673–4498 calBCE at 2σ (5730 ± 15 BP; UCIAMS-223201)], was found in the middle stratum with bones of mostly wild animals (saiga antelope, aurochs, and horses assigned to *Equus ferus*) but with early occurrence of domesticated sheep-goat bones (13% of the bones identified to species). This sample was plotted in the NEONCAS cluster [(*2*), extended data: figure 5a] with wild horses a millennium older. Either the Eneolithic horse population in the Pontic-Caspian steppes varied between NEONCAS and Ukr11, or a genetic shift occurred between ∼4600 BCE at Oroshaemoe and ∼4200 BCE at Semenivka.

Yamnaya horses in the DOM2 clade (hereafter referred to as DOM2-clade) from Repin on the Don [3265–2913 calBCE at 2σ (4400 ± 30 BP; UCIAMS-218275)] labeled CPONT and Turganik in the Volga-Ural steppes [2889–2680 calBCE at 2σ (4195 ± 20 BP; UCIAMS-199647)] labeled TURG, and a DOM2-clade Steppe Maikop horse from a kurgan grave at Aygurskii in the Manych steppes between the Don and Volga [3526–3373 calBCE at 2σ (4695 ± 20 BP; UCIAMS-218225)], were among the direct ancestors of modern DOM2 horses dated after ∼2200–2100 BCE (hereafter referred to as DOM2-mod). Their genetic contribution to DOM2-mod was “approximately 95%” [(*1*), p. 2]. Hosek *et al.*’s (*4*) claim that there was no connection between Yamnaya horses and modern domestic horses ignored this conclusion. The “where” question for DOM2 ancestry was answered by Librado *et al.* (*1*): the Pontic-Caspian steppes. However, the “when” is debated, as well as the role of riding.

In a very recent paper from the Orlando group (*3*), Liu *et al.* referred to such indicators of human management among early DOM2 horses as “early taming efforts.” They made date estimates for the beginning of early taming, or what we consider the early domestication process, by modeling the number of generations ago that selection began at the two key loci that showed the earliest selective pressure: GSDMC (back endurance, front leg strength) and ZFPM1 (fear, anxiety). Beginning dates tended to be underestimated by their methods, as they warned. Selection began on ZFPM1, the earliest indicator, as early as ∼3500–3400 BCE (680 generations ago × 8 years per generation), but the modeled date was 3010 BCE. Selection on GSDMC, a more direct indicator of riding, was almost as early. Both loci began to undergo intensified selection beginning between 3064 and 2564 calBCE, modeled as ∼2700 BCE, extending to ∼2100 BCE. The period between ∼2700 and 2100 BCE was a “domestication bottleneck” in horse genetic diversity when horse populations lacking these traits were increasingly abandoned, reducing the effective population size of DOM2 horses. This implies that selection-inducing riding of DOM2 horses began before ∼2700 BCE, during or possibly before the Yamnaya period.

## DISCUSSION

The recent papers by Librado *et al.* (*2*) and Hosek *et al.* (*4*) discuss five topics related to how DOM2-clade horses were or were not used before ∼2200–2100 BCE. The topics are (i) DOM2-clade ancestry in CWC horses, (ii) DOM2-clade ancestry in the Altai and in Central/Southeast European horses, (iii) equine milking, (iv) genetic evidence for controlled breeding, and (v) human skeletal adaptations and pathologies associated with riding.

### DOM2-clade ancestry in CWC horses

The indirect genomic evidence against Yamnaya horse riding presented first by Librado *et al.* (*1*) and the principal topic of Librado *et al.* (*2*) was that the horses of the CWC (∼2925–2100 BCE) in Central and North Europe had no steppe horse (DOM2-clade) ancestry, and therefore, the migrations that conveyed 75 to 100% steppe-derived ancestry to European CWC human populations (*6*–*8*, *17*) were not aided or driven by DOM2 horseback riding. Librado *et al.* (*2*) used the same acronym, DOM2, for what here are DOM2-clade (including Yamnaya horses) and DOM2-mod (after ∼2200–2100 BCE). They reasoned that steppe riders would have brought their DOM2 horses with them if they rode DOM2 horses in the steppes, and the absence of DOM2 ancestry in the CWC horses from a single site in northern Bavaria (Franconia), Germany (described below) demonstrated that neither Yamnaya nor CWC people rode horses with DOM2 ancestry.

**Fig. 8.**
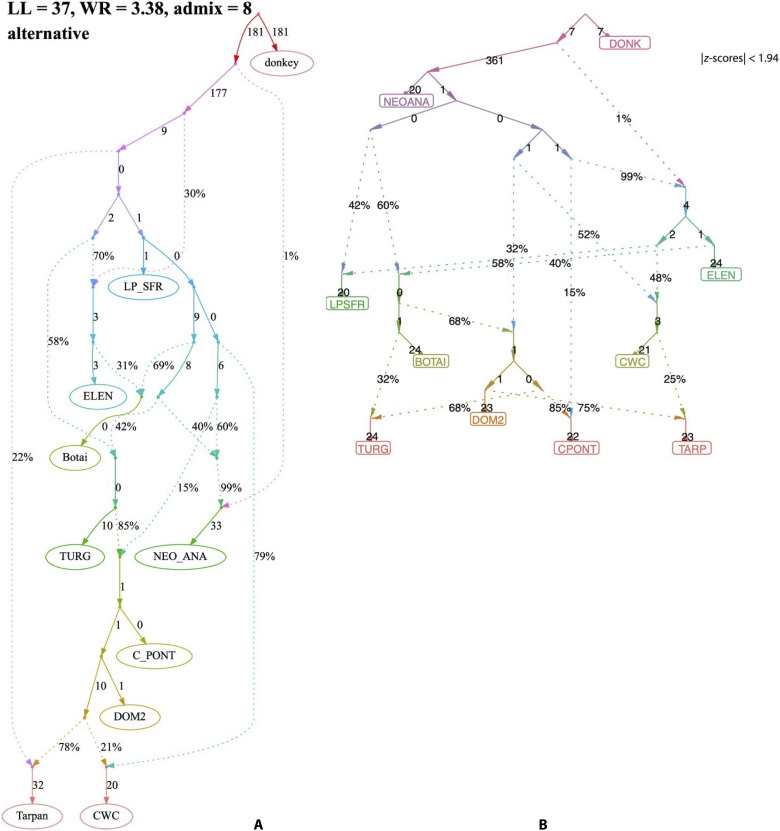
Two contrasting models for CWC horse ancestry. (**A**) With 21% DOM2 ancestry based on modeling by Maier *et al.* Reproduced from (*14*), figure 3b, under a CC BY 4.0 international license. (**B**) With no DOM2 ancestry based on modeling by Librado *et al.* LL, log-likelihood; WR, worst f4-statistic residuals. Reproduced from (*2*), extended data: figure 3b, under a CC BY 4.0 international license.

Maier *et al.* (*14*) reanalyzed Librado *et al.*’s (*1*) genetic data and showed that admixture graphs made with different tools and algorithms might show ~21% DOM2-clade ancestry in the four sampled CWC horses (Fig. 8A). Their models fit Librado *et al.*’s (*1*) data better than the published model with no DOM2 ancestry. Librado *et al.* (*2*) reanalyzed their data using similar tools (ADMIXTOOLS 2) to Maier *et al.* (*14*) with 124 newly genotyped horse samples but no new CWC samples and again found no DOM2 genetic contribution into the sampled CWC horses (Fig. 8B).

Maier *et al.* (*14*) found that many different admixture graphs fit the 2021 (*1*) data [80% of the 2024 (*2*) individuals] almost equally well. In their 10 best-performing models, four showed no DOM2 contribution to CWC, as Librado *et al.* (*1*) found, but six supported varying amounts of steppe admixture in CWC horses. All 10 models were said to fit Librado *et al.*’s data better than their published 2021 (*1*) graph, and none could be excluded. For this reason, Maier *et al.* (*14*) warned that “Graphs modeling more than six populations [Librado *et al.* (*2*) used 14 populations] and two or three admixture events [both Maier *et al.* (*14*) and Librado *et al.* (*2*) got the best fits with eight admixture events] are often not unique, with many alternative models fitting nominally or significantly better than the published one. Our results suggest that strong claims about population history from admixture graphs should only be made when all well-fitting and temporally plausible models share common topological features.”

Two topological features were shared by all well-fitting graphs in the 2021 (*1*) data: (i) DOM2-mod and its direct ancestors CPONT and TURG (Yamnaya horses) were in the same clade in all models; and (ii) Anatolian Neolithic horses had ancestry from a deeply archaic “ghost” population, not relevant here. The topological features related to DOM2 ancestry in CWC horses varied between models, so strong conclusions about this topic cannot be supported until a clearer signal can be generated from a larger sample of CWC horses.

The four CWC horses reported by Librado *et al.* (*1*, *2*) all came from one unusual site, Hohler Stein at Schwabthal (town of Bad Staffelstein, Upper Franconia), identified as a CWC “settlement” in their supplementary tables, but described in publications as rather a ritual site containing mixed deposits of pottery, animal bones, fires, and a few human bones including adults and fetuses, with more Neolithic LBK than CWC pottery but also including Middle Bronze Age and later ceramics (*51*). The almost identical radiocarbon dates of 2860–2502 calBCE at 2σ (4095 ± 20 BP, 4115 ± 20 BP, and 4090 ± 20 BP; UCIAMS-224892-4) on the horse bones suggest an event of the CWC era, likely associated with the CWC pottery at the site. The tight radiocarbon dates suggest that it might have been a single event. Horses or their parts selected for a single Franconian mountain ritual with Neolithic origins cannot represent all CWC horses. CWC horse remains are frequently mentioned in the otherwise rare CWC settlements in Central Europe (*52*, *53*), like in the only 5-km distant, roughly contemporary, and better published site of Wattendorf-Motzenstein (*54*), or the CWC lake-side dwellings of Switzerland [(*55*), p. 110–112]. However, they are always recorded in low specimen numbers, often only one or two individuals (*56*). In this respect, CWC horse bone occurrence pattern is not dissimilar to the earlier, ∼3500–3000 BCE Central European horse horizon. Like before, they never show up in CWC graves, unless it is a tool made of horse bone (*57*). This all speaks for horses of this period being more of a precious prestige good than the usual herd animal. The finding that Yamnaya CPONT horses were in the same clade with DOM2-mod horses has stronger support than the claimed absence of DOM2 ancestry in CWC horses, which itself is at best an indirect indicator of whether Yamnaya people rode DOM2-clade or any other kinds of horses.

### DOM2-clade ancestry in horses in the Altai and Central/Southeast Europe

No horse from a Yamnaya, CWC, or Afanasievo grave has yielded useful aDNA except for one Afanasievo coat color allele described below. Horse bones were found in the fillings of at least five Yamnaya kurgans in Southeast Europe, as described in Supplementary materials 1 of (*16*), but none were analyzed for aDNA. In their effort to determine whether steppe horses expanded with steppe people, Librado *et al.* (*2*) filled this data gap with scattered horse ancestry data from before and after the Yamnaya expansion in Central and Southeast Europe. Hosek *et al.* [(*4*), p. 2] denied that domestic horse bones occurred in any Afanasievo archaeological context, arguing that Yamnaya people migrated to the Altai on foot or in ox wagons, without horses.

#### Horses in the Altai

The Afanasievo culture (∼3100–2600 BCE) was a pastoral culture broadly similar materially and in burial customs to Yamnaya, but it appeared 2000 km to the east in the Altai Mountains, western Mongolia, Xinjiang, and the Minusinsk basin (*58*), with only a handful of related sites in the intervening steppes of central Kazakhstan. Human aDNA from Afanasievo graves showed that their ancestry was derived almost entirely, more than 98%, from the Core Yamnaya population that lived west of the Ural River, although a few individuals had some western Siberian admixture (*7*, *8*). It is accepted (*59*, *60*) that the Afanasievo migration introduced kurgan graves, copper metallurgy, wheeled vehicles, and multispecies pastoralism (cattle, sheep-goat) to the Altai. Afanasievo sheep bones from the Nizhnyaya Sooru settlement directly dated ∼3100–2900 BCE (3346–2883 BCE at 2σ; 4460 ± 40 BP and 4320 ± 20 BP; Poz-113280-81) showed genetic ancestry from western Asian domesticated sheep with the unusual mt-haplogroup D [(*61*), p. 3], so Afanasievo migrants introduced western Asian sheep to the Altai. Did they also bring horses?

Kuzmina [(*62*), p. 205] identified nine Afanasievo kurgan cemetery sites in the Altai that produced horse bones: Chernovaya VI, Letnik VI, Krasny Yar, Malyie Kopani, Tepsei X, Kuyum, Bike, and Elo. These show that horses were sacrificed in some Afanasievo funerals, like some Yamnaya funerals in Southeast Europe. At four Afanasievo settlements in the Altai—Nizhnyaya Sooru, Kara-Tenesh, Malyi Dugan, and Balyktyiul’—the percentage of horse bones varied from 1 to 9%, averaging 5% in a pastoral economy dominated by sheep-goat (average 79%) and cattle (12%) [(*60*), p. 4]. At Nishnyaya Sooru, the Afanasievo settlement was not reoccupied later, so the horse bones must be Afanasievo in age. Denisova Cave contained in layers 11 and 12 an Afanasievo occupation with diagnostic ceramics and stone-lined hearth features, and a horse bone from that occupation (*63*) (BER001, labeled in table S5 as domesticated) had the allele for chestnut coat color, found in domesticated horses and not in Pleistocene or early Holocene wild horses. DOM1 Botai horses had earlier exhibited coat colors associated with domesticated horses (tobiano spotting, “silver”) (*9*), so the Afanasievo chestnut color was not isolated. Zoologist Kosintsev (*64*) reserved his opinion on whether Afanasievo horses were wild or domesticated, noting that the available zoological measures were insufficient, but horses were consistently present in Afanasievo settlement faunas with sheep-goat and cattle, and appeared occasionally in graves.

Aside from the chestnut coat color allele from Denisova Cave, no Afanasievo horse aDNA is published. The oldest-dated horse genetic sample with a published assignment to a DOM lineage from the wider Altai–Sayan–western Mongolia region at this time is an upper P_2_ tooth from a DOM1 horse that plots near Botai horses in figure 4 of (*65*), dated 1871–1643 BCE at 2σ (3430 ± 15 BP; UCIAMS268488). It was recovered from the Tsengel Khairkhan glacier in the Mongolian Altai, an inhospitable environment for horses, so probably was brought there by humans, perhaps as a skull for ritual placement. We cannot yet assign a DOM lineage to Afanasievo horses in the Altai dated more than a millennium earlier. Botai-related DOM1 horse eaters (with evidence for corralling, milking, and riding, as reviewed above) were still present across the northern Kazakh steppes in ∼3100–3000 BCE when a subpopulation of Core Yamnaya DOM2 horse-keeping pastoralists (likewise with evidence for milking and riding) abandoned the Pontic-Caspian steppes and migrated 2000 km to the Altai, where the cultural innovations that defined Afanasievo material and burial culture thereafter were unexpectedly rapidly adopted by their emerging culture. Both DOM1 and DOM2-clade horses might be expected in this situation, particularly if their behavior and aptitude for riding was similar during this early phase of domestication.

Hosek *et al.* [(*4*), p. 2] asserted in reference to Afanasievo faunas in the Russian Altai and western Mongolia that “…the archaeofaunal record in the region shows no evidence so far that they used, raised, or even brought domestic horses with them… which have yet to be identified in the region before the late second millennium BCE.” The qualifier “domestic” makes that statement mostly accurate, but Afanasievo people certainly used horses. Honeychurch and Orlando [(*66*), p. 27] accepted that Afanasievo people regularly used horses but concluded that they were “in all likelihood wild.”

We tend to disagree. No morphological or frequency data support a choice between wild and domestic for Afanasievo horses, as the zoologist Kosintsev noted (*64*). The chestnut color allele from Denisova Cave might suggest domestic but needs additional genetic support. Archaeological and human genetic data from the Altai show that horses were one element in a package of innovations that introduced domesticated western Asian sheep, goats, cattle, and arguably horses, with wagons, copper metallurgy, and kurgan cemeteries. Most Altai Afanasievo sites were unoccupied before the Afanasievo period so the presence of wild horses in the high Altai at the time of the Afanasievo migration is not well established. An exception is Ust-Biyke-I, but there is a 3000-year gap between the dated Afanasievo occupation and the Late Mesolithic, and the fauna is highly fragmented (*59*). Svyatko *et al.* (*67*) concluded that domesticated horses probably were present in Afanasievo herds with cattle, goats, and sheep, but more conclusive evidence is needed.

#### Horses in Central and Southeast Europe

The expansion of DOM2-clade Yamnaya horses westward toward Europe was a principal topic of Librado *et al.* (*2*). They asserted that DOM2 horses appeared in the Carpathian basin only after ∼2200–2100 BCE, long after the Baden horses and Yamnaya migrations, supporting their contention that Yamnaya migrants did not ride DOM2 horses: “Combined, these findings narrow down the time for the genomic turnover accompanying the arrival of DOM2 horses in the Carpathian basin to ~2033–1945 BCE, …post-dating the arrival of human steppe-related ancestry in the respective regions by at least 600 years. Yamnaya-related steppe migrations and the spread of DOM2 horses are, thus, chronologically incompatible.”

DOM2 here means DOM2-mod horses, which did not exist before ∼2200–2100 BCE, so it is not indicative that earlier Yamnaya populations did not have them. The Yamnaya migrations into Southeast Europe that began ∼3100 BCE (*8*) were compatible with the spread of DOM2-clade horses, as Librado *et al.* (*2*) recognized. For that reason, they analyzed the genetic contribution of DOM2-clade horses to Indigenous European (DOM3) horse populations in Central and Southeast Europe. However, geographic patterning in that admixture is unclear, as they located some sites incorrectly and did not attempt to compare closer to the steppe with more distant populations. Chronological patterning is obscured because they were not able to divide their sample into pre-Yamnaya and post-Yamnaya groups. Last, they did not discuss the archaeological/zoological debate about the arrival of steppe horses in Southeast Europe, as interpolated here.

Librado *et al.* (*2*) first combined all DOM2-clade steppe horses (CPONT, TURG, and NEONCAS) into one DOM2-clade group. *F*_4_ statistics indicated that this source accounted for 55% of the ancestry in four lower Danube valley horses from two well-known Eneolithic Gumelnitsa culture tell sites, Pietrele and Căscioarele, dated 4494–4355 and 4342–4251 calBCE at 2σ (5600 ± 30 BP; UCIAMS-218288 and 5435 ± 20 BP; UCIAMS-199654), respectively, on the analyzed horse bones. They mistakenly placed both sites in Transylvania and claimed that they had no steppe contact. However, these sites were in the lower Danube valley and contained imported steppe-derived pottery [(*68*), pp. 378–391, figures 10 to 16]. Not remarked by Librado *et al.* (*2*), one Pietrele horse carried an MC1R allele for chestnut coat color (*69*), dated earliest at this site, perhaps introduced with its predominant DOM2-clade ancestry. These tell sites were contemporary with steppe-derived human populations who migrated to Giurgiulesti in the lower Danube valley, to Targovishte (Gonovo mogila) in the Thracian plain, and to Csongrád in the Carpathian Basin (*8*) at ∼4500–4200 BCE (*70*–*73*), after which all tell settlements in the lower Danube valley, including Pietrele and Căscioarele, were burned and abandoned and their cultures ended (*74*–*76*). The Csongrád steppe migrant, who came from a Caucasus–Lower Volga steppe population (*8*), had skeletal adaptations and pathologies consistent with habitual horseback riding (*16*) (see likewise below), so 55% DOM2-clade horse ancestry in Eneolithic horses in the lower Danube valley could have been related to horse riding long before Yamnaya.

In the Carpathian Basin and Central Europe, farther from the Pontic steppes, most of the sampled horses had less DOM2-clade ancestry. It was not possible to compare pre-Yamnaya and post-Yamnaya horse ancestries to see how they differed; instead, Librado *et al.* (*2*) combined all sampled horses from the Carpathian Basin dated between 3364 and 1971 calBCE into one group. Most of these were from the Csepel Island Bell Beaker contexts in Hungary dated ∼2400–2200 BCE but some were pre-Yamnaya horses. They found 17.2% DOM2-clade ancestry in this combined group. Variation in the group was not described. In their online catalog, the 3364 BCE date is associated with a horse from the TRB and Boleráz culture site of Stránská skála, Czechia (at the edge of the Carpathian Basin), dated 3364–3108 calBCE at 2σ (4540 ± 15 BP; UCIAMS-218463), possibly from a specimen discussed as a possible import from the steppes because of its unusually large size, 157 to 168 cm at the withers [(*37*), p. 315]. The bar graph of the ancestry components of the Stránská skála sample by Librado *et al.* [(*2*), figure 1b] includes no DOM2-clade ancestry, so it seems to suggest that its large size was not associated with steppe ancestry, an important result if this is the same horse. However, another horse from a Baden settlement at Kittsee, in Burgenland, eastern Austria, dated 3371–3109 calBCE at 2σ (4560 ± 20 BP; UCIAMS-278407), had 28.9% DOM2-clade ancestry. This indicates that some Baden horses, which were on average notably larger than earlier horses in Southeast Europe (see again our Fig. 5), had some DOM2-clade ancestry, and some of this could have come from steppe horses introduced by Csongrád-like people in the Eneolithic or possibly by pre-Yamnaya people (*71*, *72*) of ∼3500–3000 BCE before the actual Yamnaya expansion, when the Yamnaya human population remained mostly in the steppe. However, the larger size and variability of Baden horses seem to have resulted largely from the independent local management of Indigenous European horses. Size variation in horses is more easily manipulated than zoologists realized (*77*). Nevertheless, the increase in variation and size after ∼3500 BCE suggests intentional interference.

Librado *et al.* (*2*) recognized that DOM2-clade ancestry in Southeast Europe dated before ∼2200–2100 BCE could have been associated with Yamnaya or earlier migrations from the steppes, but they argued that many European horse populations including a Paleolithic horse from France dated to the mid-15th millennium BCE showed traces of ancestry from a deep ancestor of DOM2-clade steppe horses. Their best-fitting admixture graph showed the steppe ancestry in the Pietrele and Cáscioarele horses coming from this hypothetical Paleolithic proto-DOM2 ancestor, contradicting their statement earlier in the same paragraph that a pooled combination of NEONCAS, CPONT, and TURG samples dated after ∼5500 BCE accounted for the same ancestry. Their conclusion that “…the spread of steppe-related horse genetic ancestry into Europe must predate ~14,646 BCE” is not well supported.

The increase in horse sizes and variability after ∼3500 BCE in Central Europe seems to reflect local management of a largely Indigenous horse population that was finally exploited intensely (45 to 60% of fauna) at Bell Beaker sites on the Csepel island near Budapest just before the appearance of the DOM2-mod mutations (*78*, *79*). Some Eneolithic and/or Yamnaya people might have ridden DOM2-clade horses into Southeast Europe but that topic requires investigation of horse aDNA from intrusive Eneolithic (Giurgiuleşti or Cernavoda I), pre-Yamnaya and Yamnaya graves. During ∼3500–3000 BCE, people in Southeast Europe experimented with horse management and therefore probably rode horses as an innovative aid to light transportation and herding, while wheeled vehicles were beginning to appear as an independent but complementary innovation in heavy transport (*39*, *80*, *81*).

### Equine milking

If Yamnaya-culture people milked horses, then domesticated mares were present in Yamnaya herds and their foals were nearby to help release their milk. Wilkin *et al.* (*15*) found distinctive peptides from horse milk in the dental calculus of two Yamnaya individuals from the Kriviansky IX kurgan cemetery in the lower Don steppes. Librado *et al.* (*2*) dismissed the relevance of this evidence: “…Equine milk peptides were reported in Yamnaya human dental calculus ~3300–2600 BCE, but further work has revealed that western steppe pastoral practices shifted from sheep and cattle dairying to horse milking no earlier than ~1000 BCE…”

The two Yamnaya samples with equid milk peptides reported in Wilkin *et al.* (*15*) are well dated (below) and valid; no further work was done on them. Scott *et al.* (*82*) did not reveal a shift to horse milking no earlier than 1000 BCE but found only one positive horse milk signal dated to the Iron Age. They (*82*) examined four Yamnaya individuals from the North Caucasus steppes and found none with horse milk peptides in their calculus; Wilkin *et al.* (*15*) examined 16 Yamnaya individuals from the Volga-Don steppes and found two with horse milk. The dates associated with the two horse milk drinkers were minimally centuries apart, indicating a continuing custom in the lower Don steppes: Krivyanski IX, Kurgan 4, grave 21: 3345–3096 calBCE at 2σ (4495 ± 25 BP; PSUAMS-7979) and Kurgan 2, grave 2: 2881–2633 calBCE at 2σ (4165 ± 25 BP; PSUAMS-7978). They may actually be earlier than stated as the dates given use a reservoir correction not recommended for the region. Overall, proteomic hits and misses are perhaps more related to preservation than actual proportions of consumption. The method is certainly more reliable in demonstrating presence than absence given preservation issues and small sample sizes [(*9*), p. 8]. So, it seems that most Yamnaya people did not drink horse milk, but some did in the lower Don valley, also home to a distinctive Lower Don Yamnaya human genetic variant population who rarely migrated (*8*), unlike the wide-ranging Core Yamnaya people, who perhaps did not milk their mares.

### Controlled breeding of DOM2 horses

Librado *et al.* (*2*) developed a method to calculate generation times in horses and observed that in the past 200 to 300 years, the average generation time for modern horses has declined to 4.1 years, compared to an average 7.4 years before the modern era. Between ∼2200 and 2100 and 1000 BCE, they observed a similar decline in generation times to average 3.5 years, which they associated with the high demand for DOM2-mod chariot horses. They plausibly equated shorter generation times with increased demand and production, but also with “new practices of DOM2 reproductive control,” which they claimed were “a prerequisite to early DOM2 breeding and adoption of widespread horse-based mobility.”

Shorter generation times might well equate with more intensive production, but not necessarily with control over breeding. In the modern sample they described, the most desired and controlled breeds—Thoroughbreds and Quarter-Horses—had the longest generation times, close to the premodern average. Demand for higher volume is not a prerequisite for controlled breeding or for widespread horse-based mobility, but it could be a result. The absence of a similar episode of declining generation times in Yamnaya DOM2-clade horses could be interpreted to indicate that the demand for CPONT horses remained steady during the Yamnaya period, while the evidence for a sharp decline in Botai generation times, a genetic signal of intense management, might have been related to the horses’ central role as meat producers in the human diet.

Indirect evidence for controlled breeding might be contained in genetic data from the early phase of domestication in the DOM2 clade. Clustering in MDS analysis indicate a large difference between the wild Volga-Don steppe horses grouped as NEONCAS, found primarily in hunter-gatherer sites dated ∼5500 BCE, and CPONT samples of the Yamnaya era, shifted toward DOM2-mod values, dated after ∼3500 BCE (see again Fig. 7). As noted above, Liu *et al.* (*3*) found that selection began on the genetic loci controlling fear and back strength during or before the Yamnaya era when CPONT genomes shifted toward DOM2-mod values in MDS. Together, these methods show that Yamnaya horses were under selection for traits that facilitated riding and handling, suggesting some control over breeding.

### Human skeletal adaptations and pathologies associated with riding

Berthon (*83*) and Berthon *et al.* (*84*) reviewed the human skeletal indicators of habitual horse riding among Avars (6th to 7th century CE) and in general, respectively, and Trautmann *et al.* (*16*) described six osteological indicators that together defined a syndrome (“horsemanship syndrome” or “horse-rider syndrome”) associated with habitual horse riding. Bareback horse riding is biomechanically very specific and almost unique in requiring many hours spent squeezing the thighs and knees together with the legs spread wide apart while bouncing vertically on a firm seat and occasionally falling, usually backward, from a moving platform above chest height. The six skeletal markers capturing these riding stresses included entheseal stress reactions at muscle attachments on pelvis and femur, acetabular ovalization, femoro-acetabular alterations, changes in femoral bone shaft cross-sectional shape, stress-induced vertebral degeneration resulting from repetitive vertical impacts, and trauma from accidental falls. No single trait was deemed sufficient to diagnose riding; it was the combination of three or four traits (or more) that presented a case increasingly difficult to explain through nonriding behaviors. The six criteria were applied to 217 mostly “steppe” individuals from 39 sites in Southeast Europe dated between fifth and second millennia BCE, of which ~150 were archeologically assigned to the Yamnaya culture. Trautmann *et al.* were familiar with rider adaptations and pathologies in a large collection of Avar horsemen (*83*) but had not expected to find similar traits in Yamnaya individuals. Nevertheless, five Yamnaya individuals from kurgan graves in Romania, Bulgaria, and Hungary dated 3021–2501 calBCE at 2σ had four or more “rider” traits, as did two pre-Yamnaya individuals from Hungary [Csongrád, dated 4442–4243 calBCE at 2σ (5470 ± 40 BP; Poz-41865)] and Romania [Blejoi, 3331–2927 calBCE at 2σ (4437 ± 34 BP; DeA-8814)]. Fifteen individuals including nine Yamnaya males presented three traits. Because the relative number of “riders” in the adult population was probably higher than that represented by the preserved cases, Trautmann *et al.* estimated that around 20% of the analyzed Yamnaya population rode horses habitually, and all who presented rider syndrome were males, while males outnumbered females in the “steppe” sample population 2:1.

Hosek *et al.* (*4*) argued that a literature review of the six osteological pathologies showed alternate explanations for each of them. Trautmann *et al.* (*16*) had previously warned that each trait had alternate possible causes and was insufficient by itself. Not remarked by Hosek *et al.* (*4*), Trautmann *et al.* (*16*) described solutions for narrowing the cause of each pathology to riding. For example, they included as possible riders individuals with strongly pronounced (at least grade 2 on a scale of 0 to 3) muscular attachment sites (entheses) for muscle groups with limited everyday biomechanical strain but high demands when riding (mostly adductors of the thigh), especially when combined with a marked femoral antetorsion. They excluded individuals with a generally high mechanical load where upper body or lower leg entheses were more pronounced, narrowing their analysis to individuals with thigh muscle development not correlated with general muscle development. Hosek *et al.* (*4*) asserted that ascribing thigh adductor development to riding was “cherrypicking” because hockey and soccer players are described in some reference works as exhibiting similar traits. However, in soccer or hockey athletes, thigh adductor development is one element in generally high muscular development, particularly in the lower legs, which would cause their exclusion using Trautmann *et al.*’s (*16*) screens. For another trait, Hosek *et al.* (*4*) suggested that habitual squatting might replicate the changes in the acetabulum attributed to rider syndrome. However, squatting facets on the tibia were almost ubiquitous in the examined Yamnaya population, yet only a few individuals displayed ovalization of the acetabulum like that seen among habitual riders. Last, the core of Trautmann *et al.*’s method was to include as riders only individuals exhibiting four traits or more, while scoring as “possible” those exhibiting three traits, so a list of alternate causes for each trait does not address the diagnosis of a multitrait syndrome.

**Fig. 9.**
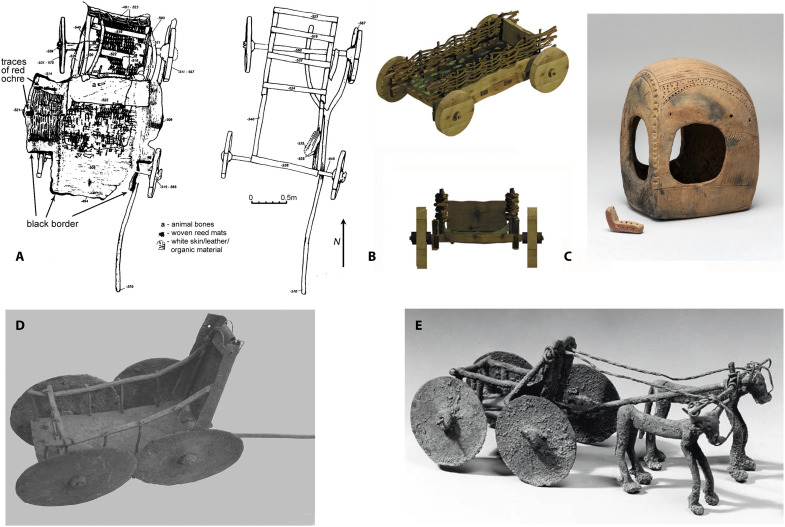
Wagons in the third millennium BCE record. (**A**) A Yamnaya-era wagon from Ostannii in the Kuban steppes, Russia; drawings from (*86*), figure 55, and (*70*), figure 4.5, used here with permission from Princeton University Press, from “The Horse, the Wheel, and Language: How Bronze-Age Riders from the Eurasian Steppes Shaped the Modern World, by D. W. Anthony, book, first edition, 2010 (permission conveyed through Copyright Clearance Center, Inc.). (**B**) A late Katacombnaya wagon reconstruction from Ulan (site IV, kurgan 4, grave 15) in the Manych steppes, Russia; from (*87*), figure 7, reproduced with permission from Cambridge University Press (permission conveyed through Copyright Clearance Center/RightsLink). (**C**) Early Katacombnaya wagon models from Chograi VIII (left) and Elista (kurgan 5, grave 8; right) in the Kalmykian steppes, Russia. Photo by N. Shishlina in the State Historical Museum in Moscow. (**D**) Bronze wagon model from Şanliurfa, Türkiye; reproduced from (*90*), figure 6, under a CC BY 4.0 international license. (**E**) Unknown location in Anatolia, Türkiye in the Metropolitan Museum New York, USA; www.metmuseum.org/art/collection/search/325825 (accessed 2 April 2025).

Hosek *et al.* (*4*) also argued that wagon or chariot riding could have caused some of the bundled pathologies seen in Yamnaya individuals. Chariots were not invented until after the Yamnaya period, so these are not relevant. Their figure 1 showing the pelvic positioning of a wagon driver had him/her sitting on a protruding floorboard with feet hanging loose, a position too low to see over the ox team. Wagon drivers either walk beside the team with a whip or goad or sit on a high box in the front of the wagon where they can see over the team to the terrain in front, a critical need in a world without roads (*85*). Did ~3500–2200 BCE wagons have a seat or high box? An overview (Fig. 9) from reconstructions of wooden wagon/cart remains from both Yamnaya and Katacombnaya graves (*86*–*88*), of Katacombnaya clay models from the Caspian-Pontic steppe, and contemporary Anatolian models in clay and bronze (*89*, *90*) finds none. Such features are likewise unknown in ~3500–2200 BCE wagon models from the Carpathian basin (*39*, *80*). Irrespective of the fact that repeatedly sitting on a wagon seat box for long periods has not been demonstrated to cause the suite of changes to the femur and pelvis seen in Yamnaya individuals with rider syndrome [and are not to be expected from a biomechanical point of view; (*84*)], such seating boxes seem not to have existed anyway. Hosek *et al.*’s (*4*) figure 1 is therefore obsolete.

**Fig. 10.**
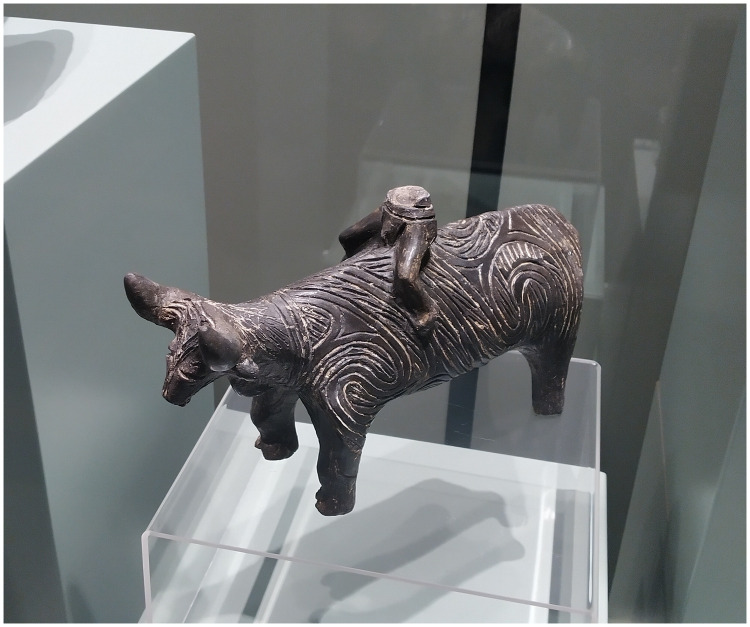
A human/boy riding a bull/ox. Ceramic figurine of ∼25-cm length from the Kapitan Andreevo site in southeast Bulgaria, dated to ∼5000 BCE (*92*); photo by V. Heyd taken in the National Archaeological Museum Sofia, Bulgaria.

Bukalov (*91*) speculated that “horse-rider syndrome” could have been caused by people riding cows, bulls, or oxen (with the very beginnings of castration being another question). People do occasionally ride cattle, and indeed a ceramic figurine of a human/boy riding a bull/ox was found at the Kapitan Andreevo site in southeast Bulgaria, dated as early as to ∼5000 BCE (*92*) (Fig. 10). However, cattle have broad backs that make it difficult for a rider to squeeze their knees together for a firm hold, and accordingly, the bull/ox rider’s seat and leg position would be very different from horse riders. Their backs are weaker and less able to tolerate sustained weight carrying than horses—cattle are rarely used as pack animals. Also, cattle are slower, clumsier, and more belligerent than horses because their first response to a threat is to fight rather than flee, so they must be selected and trained like horses, and their transport benefit is much less. Occasional short-distance rides from farm to field, or in ritual contexts, on a tolerant, patient bull/ox occurred, as Fig. 10 demonstrates, but habitual riding over many years of the kind that causes horse-rider syndrome is not known to occur among cattle keepers.

Trautmann *et al.*’s (*16*) method has not yet been applied to Yamnaya skeletal collections in the Pontic-Caspian steppes. Buzhilova (*93*) analyzed changes in entheseal development in elbows and knees, not certainly associated with riding, and quite different from Trautmann *et al.* However, horsemanship syndrome was identified (*94*) using Trautmann *et al.*’s method in a population of the post-CWC Strzyżów culture in southeast Poland dated ∼2000–1600 BCE, where seven of 23 measurable individuals, including three females, had three pathologies associated with riding and four had four traits, which the authors accepted as evidence for the practice of horse riding by both sexes in that culture. If applied correctly, Trautmann *et al.*’s method seems to identify habitual riders by changes to the upper thigh, pelvis, and lower back. By these standards, some Yamnaya people, all or mostly males, probably were habitual riders.

If some Yamnaya people rode horses habitually in Southeast Europe, it is reasonable to assume that they also did in the Pontic-Caspian steppes, where horses had played an important symbolic role in human rituals and prestige displays (maces) and an important economic role as sources of food as early as ∼4500–4200 BCE at Khvalynsk (*49*), in ∼3500–3100 BCE at Botai (*9*), and in ∼3200–2600 BCE in Yamnaya (*22*, *25*, *27*, *28*) and possibly Afanasievo (*59*–*66*) contexts.

To sum up, some horse riding is necessary to manage horses in the open, and the highly mobile form of pastoralism invented by the Yamnaya culture arguably required mounted herders particularly in the drier steppe/semi-desert of the Caspian-Manych and Azov steppe regions (*28*, *33*). A mounted herder in Mongolia can control three times more sheep than a pedestrian herder [(*95*), p. 32]. The increase in herd sizes permitted by mounted herding produced a surplus that could be used in feasting [such as that occurred at Khvalynsk; (*49*)] and gift-giving. When horse riding was combined with ox-drawn wagons ∼3200 BCE, the domestic unit became mobile and an innovative kind of pastoral economy was invented, opening the vast bioenergy of the Eurasian steppes to human exploitation and giving birth to the Yamnaya culture (*70*, *96*, *97*).

Although horses that belonged to the genetically distinct Central Asian (DOM1) and European (DOM3) populations probably were ridden also ∼3500–3100 BCE, the economic context of riding was different in each region. It was only in the Pontic-Caspian steppes that riding led to selection for DOM2-mod traits, perhaps because the mobile, multispecies pastoralism that evolved in the western steppes required longer work hours from more horses than did other economies, increasing the pool of worked horses and the intensity of selection in the DOM2 clade. In the 19th century in the North American plains, both “Cowboy” and Indigenous riders who rode horses daily for herding work needed to maintain at least six to eight riding horses if they ate only grass, resting them frequently to maintain their strength [(*85*), p. 102].

Early riding would have had important social, economic, and political effects. The Yamnaya population engaged in rapid, long-distance migrations on a larger scale than any agricultural or pastoral Eurasian population had done before. Their CPONT and TURG horses were in the DOM2 clade, contributed 95% of DOM2-mod ancestry, and were shifted in MDS toward the DOM2-mod gene cluster implicated in riding. The decline in archaeologically documented settlements between the Eneolithic period and the Yamnaya era indicates increasing residential mobility in the steppes, partly attributable to horse riding. Yamnaya horses probably were more “skittish” than DOM2-mod horses, and Yamnaya riders might well have dismounted to confront a violent, messy, or noisy threat. However, even if horses only served as “taxis,” with little direct military role, they played an important role in connecting places that had not been connected, and in managing herds, in sizes not seen before.

Last, we must acknowledge a broader problem that underlies these debates. Why does the material evidence for riding increase in the steppes only ∼1200–1000 BCE if horse riding began in ∼3500 BCE or earlier? Taylor and colleagues have shown (*98*, *99*) that horses became central actors in sacrificial cults in Mongolia involving the construction of stone monuments (*khirigsuurs*) and deer-stones from ∼1200 BCE, with many indicators of horse milking and riding beginning only then in that region. In China, horses and chariots appeared suddenly in the late Shang dynasty from ∼1200 BCE, introduced from the northern steppes (*100*, *101*). The earliest images in eastern Eurasia of short recurve bows with the “Cupid” or B shape appeared on deer-stones and in Shang dynasty oracle bones. The role of horses changed at this time probably because mounted archery, which had been ineffective with the long bows of the Bronze Age, became militarily effective after the invention of the composite recurve bow ∼1200 BCE, the first powerful bow short enough to use on horseback (*102*, *103*). Beginning ∼1000 BCE, these innovative bows were paired with the first mass-produced socketed arrowheads (*104*), which appeared in steppe sites distributed from the Tien Shan (*105*) to the Carpathian basin (*106*). Recurve bows gave horse riders a deadly weapon easily used on horseback while a groundbreaking arrowhead casting technology leveled arrow weights and increased the ammunition that each archer carried. These advances in bow and arrow technology led to the rise of militarily effective mounted archery at the beginning of an era of steppe nomadism that brought steppe nomads like the Scythians into economic and military relations with emerging centralized states (China, Persia, Anatolia/Caucasus, and eventually Greece), and an innovative era in warfare began. This explanation for the lateness of cavalry as a military force does not require that riding did not exist before ∼1200 BCE, but only that it was militarily peripheral, limited to leadership (generals) and support roles (messengers, herders).
